# What predicts survival in glioblastoma? A population-based study of changes in clinical management and outcome

**DOI:** 10.3389/fsurg.2023.1249366

**Published:** 2023-08-30

**Authors:** B. Fekete, K. Werlenius, M. Tisell, A. Pivodic, A. Smits, A. S. Jakola, B. Rydenhag

**Affiliations:** ^1^ Department of Clinical Neuroscience, Institute of Neuroscience and Physiology, Sahlgrenska Academy, University of Gothenburg, Gothenburg, Sweden; ^2^ Department of Oncology, Sahlgrenska University Hospital, Gothenburg, Sweden; ^3^ Department of Oncology, Institute of Clinical Sciences, Sahlgrenska Academy, University of Gothenburg, Gothenburg, Sweden; ^4^ Department of Neurosurgery, Sahlgrenska University Hospital, Gothenburg, Sweden; ^5^ Department of Neurology, Sahlgrenska University Hospital, Gothenburg, Sweden

**Keywords:** glioblastoma, population-based, survival, prognostic factors, treatment

## Abstract

**Background:**

Glioblastoma is the most common and most aggressive primary brain tumor in adults. Despite multimodal treatment, the median survival time is 15–16 months and 5-year survival rate 5%–10%. The primary goal of this study was to identify prognostic factors for survival in an unselected population of patients operated for glioblastoma. The secondary goal was to explore changes in outcome and the clinical management of this patient group over time.

**Methods:**

We identified 222 consecutive adults operated for glioblastoma between November 2012 and June 2016 at the Department of Neurosurgery, Sahlgrenska University Hospital in Gothenburg, serving a health care region in the western part of Sweden with 1.900.000 inhabitants. Clinical variables were identified and tested as predictors for prognosis in extended Poisson regression models. The results were compared with a previously published cohort from 2004 to 2008, before current standard of care based on molecular tumor diagnosis was fully implemented.

**Results:**

Median overall survival was 1.07 years, which was significantly longer than in the 2004–2008 cohort (1.07 vs. 0.73 y, age- and sex adjusted HR = 1.89, *p* < 0.0001). Variables associated with longer survival in the multivariable model were MGMT promoter hypermethylation, non-central tumor location, complete resection of enhancing tumor, WHO performance status 0–1, unilateral tumor location, fewer lobes involved, younger age and no comorbidities.

**Conclusion:**

The median survival for patients with glioblastoma treated according to current standard treatment has moderately but significantly increased, with MGMT promoter hypermethylation as the strongest predictor for survival.

## 1. Introduction

Glioblastoma (GBM) is the most common and most malignant primary brain tumor (1), accounting for approximately 14% of all CNS tumors (2). The 2016 WHO Classification of CNS Tumors introduced molecular criteria for classification and divided GBMs into isocitrate dehydrogenase-wildtype (IDH-wt), previously classified as primary GBM (about 90%), and IDH-mutant GBM, previously secondary GBM (about 10%) (1). In the recent WHO classification, based on a multilayered approach and incorporating tumor morphology, molecular characteristics and DNA methylation profiles, all IDH-wt astrocytomas, irrespective of grade, are classified as GBM (3).

Several clinical factors have been shown to affect survival of GBM at group level. Among these, patient age (4–10), functional status (4, 6–10), tumor location (8, 10–12), extent of resection (6, 7, 9, 10, 12), multifocality (5, 6), bilaterality (5, 7, 10), type of oncological treatment (5–7, 9–12), and the methylation status of the MGMT (^6^O-methylguanine-DNA methyltransferase) gene promoter (13, 14) are the strongest predictors for survival. Often, these factors have been evaluated in isolation, while in the clinical situation a combination of patient-, tumor- and treatment-related factors is typically used.

The current standard treatment for newly diagnosed GBM is maximal safe resection followed by radiotherapy with concomitant and adjuvant temozolomide (TMZ) (13, 15). Addition of locoregional treatment by Tumor Treating Fields (TTFields, Optune®) to adjuvant TMZ has shown to prolong survival (16). Despite this multimodal treatment, patients with GBM still face poor prognosis (13, 17, 18).

Overall survival (OS) as reported in the above-mentioned randomized trials is around 15–20 months. In unselected cohorts with varying proportions of elderly patients or patients with poor performance status (PS), the median OS is 10.1–12.3 months (19–22). Thus, the survival benefit achieved in a controlled setting is only to a limited extent observed in a population-based setting. In our previously published GBM cohort from 2004 to 2008, the median OS was as low as 0.73 years (8.8 months) (5). Since patient recruitment was prior to full implementation of multimodal treatment, we found it of interest to perform a new and similarly designed study in patients treated by current standard of care. The primary goal was to report outcome and prognostic variables for survival after the introduction of multimodal treatment. The secondary goal was to compare these results with previous data, as to highlight the changes in management and outcome over time.

## 2. Methods and materials

### 2.1. Patient characteristics

We performed a consecutive study with prospectively registered clinical data from patients referred to the Neurosurgical Department at Sahlgrenska University Hospital in Gothenburg. The department serves a health care region in the western part of Sweden with 1.900.000 inhabitants and all patients with radiologically suspected brain tumors are referred to multidisciplinary team conferences.

A total of 378 patients presented with a radiologically suspected GBM during the study period. Of these, 247 (65.3%) were considered suitable for surgery and thereby met the inclusion criteria for the present study. Inclusion criteria were adults (≥18 years) who underwent resection or biopsy for a supratentorial tumor and received a first-time histological diagnosis of GBM (1). The 131 (34.7%) patients not considered to benefit from surgery, who had radiological diagnosis of GBM without tissue diagnosis, have been presented in a separate study (23).

**Figure 1:**
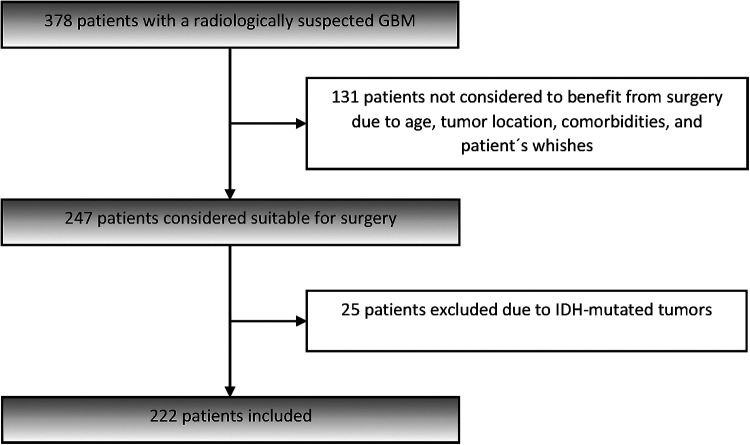
Flow chart of patient selection.

Patients were recruited from November 2012 through June 2016, included after informed consent, and followed until 30th of June 2018. Of the 247 operated patients, 25 patients had IDH-mutated tumors and were excluded from the analysis. The remaining 222 patients, 213 with confirmed IDH-wt GBM and 9 with missing IDH-status, were included in the present cohort (Figure 1). Demographics, preoperative symptoms, WHO PS as assessed by the surgeon, tumor location, presence of multifocality (defined as at least two separate contrast-enhancing tumors on MRI) and comorbidities were recorded.

One of the objectives of the present study was to investigate whether survival of GBM in our region had increased over the past decade. For comparison, we used our previous retrospective population-based study, including 229 consecutive adult patients operated at our hospital from January 2004 to December 2008, (and followed until 31st of December 2010) (5). In that study, we reported 430 patients discussed at multidisciplinary tumor conferences, of which 229 operated and included in the study. Patients with secondary GBMs met the earlier inclusion criteria. For objective comparative analysis of the two cohorts, the 25 patients with IDH-mutated tumors in the 2012–2016 cohort have therefore been included.

### 2.2. Molecular tumor diagnosis

Analysis of IDH-gene mutations and MGMT promoter methylation were performed retrospectively. Mutation analysis of IDH1 (R132H) was performed by Sanger sequencing or by immunohistochemistry as indicated (24, 25). The methylation status of MGMT promoter was analyzed by pyrosequencing using the PyroMark PCR kit (Qiagen) as previously described (26), with cut-off value ≥9% for hypermethylated MGMT promoter.

### 2.3. Treatment characteristics

Type of surgery, data on primary oncological treatment including treatment at recurrence, were recorded for all patients. Patients who underwent resection (but not biopsy) were evaluated with postoperative MRI within 72 h. The extent of surgical resection was defined as 1) complete resection of enhancing tumor (CRET), 2) incomplete resection or 3) biopsy (open or stereotactic).

### 2.4. Statistics

For descriptive purposes, continuous variables were presented by mean, standard deviation, median, minimum and maximum, and categorical variables by numbers and percentages. For test between two groups Fisher's exact test was used for dichotomous variables, Mantel-Haenszel *χ*^2^ trend tests for ordered categorical variables, *χ*^2^ test for non-ordered categorical variables and Mann–Whitney *U*-test for continuous variables. Crude event rates were calculated as number of events divided by the sum of follow-up years for a specific group of patients and expressed per 10 patient years. The 95% confidence intervals were computed using exact Poisson limits. Extended Poisson regression was used as a method of survival analysis to study interaction between various variables and time in study (27). In the baseline hazard function, time was modelled including break points at follow-up of 1 and 3.5 years. The associated survival function showed to correspond well to the estimated Kaplan–Meier survival rates. For each main effect variable, the interaction term with time was tested. For significant interaction with time (*p* < 0.05) the hazard ratios (HR) for both the main effect variable and interaction with time were presented. Otherwise, the HR was only presented for the main effect variable. Oncological treatment was analyzed in two ways: (1) Assuming the group category to be assigned already at start, that leads to introduction of a statistical error called immortal time bias. These analyses were performed to be able to compare this study's results to other studies' results, where this error was ignored. One could interpret the results as the effect of following a certain pattern of treatment (if we already at baseline knew what pattern the patient would be able to follow), but not as the effect of the future treatment/intervention *per se*. (2) Handling the treatment/intervention as time-updated variable. In this analysis, a patient is categorized into no treatment/intervention until treatment is provided, which can be interpreted as the effect of treatment/intervention *per se*. Age- and sex-adjusted models were performed, including interaction with time if needed. A multivariable model was obtained using backward selection, keeping only statistically significant variables in the model. The HRs for finally selected variables were transformed into HRs per 1 SD increase, to be able to compare the impact between variables on survival. All tests were two-tailed and significance level of 0.05 was used. Survival duration was calculated from the day of the surgery to the day of death. Patients alive at the end of the study, 30th of June 2018, were censored All analyses were performed using SAS software version 9.4 (SAS Institute Inc., Cary, NC, USA).

## 3. Results

### 3.1. Patient characteristics

The baseline clinical characteristics of the 222 patients are shown in Table 1. As shown, there were 139 men (62.6%) and 83 women (37.4%) (male-to-female ratio of 1.67). The median age at surgery was 64 years (range 19; 82 years). At the time of diagnosis, 156 (70.3%) patients had WHO PS 0–1 and 66 (29.7%) had WHO PS 2–4. Most patients had multiple presenting symptoms (*n* = 153, 68.9%). The most common presenting symptoms, either as a single symptom or as one of several symptoms, were focal neurological deficits (*n* = 142, 64.0%), headache (*n* = 90, 40.5%), and seizures (*n* = 58, 26.1%). 132 (59.5%) patients had at least one comorbidity, e.g., hypertonia, diabetes, autoimmune disease. Most frequent tumor location was temporal (51.8%), and slightly more tumors in the right hemisphere compared to the left (49.5% vs. 42.8%). MGMT promoter methylation status was available for 210 cases, in 109 of all cases (49.1%) the MGMT promoter was hypermethylated.

**Table 1:** Patient characteristics.

Variable	Total *N* = 222	Alive *N* = 18	Dead *N* = 204
Sex			
Male	139 (62.6%)	13 (72.2%)	126 (61.8%)
Female	83 (37.4%)	5 (27.8%)	78 (38.2%)
Age at operation (years)	61.9 ± 10.7	58.2 ± 11.3	62.2 ± 10.6
	64 (19–82)	59 (39–77)	65 (19–82)
Age at operation (years) (cat.)			
<50	29 (13.1%)	3 (16.7%)	26 (12.7%)
50-<60	58 (26.1%)	7 (38.9%)	51 (25.0%)
60-<70	88 (39.6%)	6 (33.3%)	82 (40.2%)
≥70	47 (21.2%)	2 (11.1%)	45 (22.1%)
Presenting symptoms			
Epileptic seizures	58 (26.1%)	6 (33.3%)	52 (25.5%)
Headache	90 (40.5%)	5 (27.8%)	85 (41.7%)
Nausea, vomiting	38 (17.1%)	1 (5.6%)	37 (18.1%)
Personality change	41 (18.5%)	3 (16.7%)	38 (18.6%)
Psychological reactions and symptoms	14 (6.3%)	1 (5.6%)	13 (6.4%)
Focal symptoms	142 (64.0%)	13 (72.2%)	129 (63.2%)
Sensory deficits	19 (8.6%)	2 (11.1%)	17 (8.3%)
Motor deficits	78 (35.1%)	5 (27.8%)	73 (35.8%)
Sight deficits	27 (12.2%)	2 (11.1%)	25 (12.3%)
Language deficits	66 (29.7%)	7 (38.9%)	59 (28.9%)
First symptoms categorization			
Incidental finding	3 (1.4%)	1 (5.6%)	2 (1.0%)
Focal deficits	33 (14.9%)	3 (16.7%)	30 (14.7%)
Epileptic seizures	14 (6.3%)	1 (5.6%)	13 (6.4%)
Cognitive	10 (4.5%)	2 (11.1%)	8 (3.9%)
Pressure	9 (4.1%)	0 (0.0%)	9 (4.4%)
Multiple	153 (68.9%)	11 (61.1%)	142 (69.6%)
WHO performance status			
WHO 0	81 (36.5%)	5 (27.8%)	76 (37.3%)
WHO 1	75 (33.8%)	9 (50.0%)	66 (32.4%)
WHO 2	42 (18.9%)	2 (11.1%)	40 (19.6%)
WHO 3	17 (7.7%)	1 (5.6%)	16 (7.8%)
WHO 4	7 (3.2%)	1 (5.6%)	6 (2.9%)
WHO 2/3/4	66 (29.7%)	4 (22.2%)	62 (30.4%)
Location			
Frontal component	87 (39.2%)	6 (33.3%)	81 (39.7%)
Temporal component	115 (51.8%)	7 (38.9%)	108 (52.9%)
Parietal component	74 (33.3%)	6 (33.3%)	68 (33.3%)
Occipital component	36 (16.2%)	3 (16.7%)	33 (16.2%)
Central component	59 (26.6%)	4 (22.2%)	55 (27.0%)
Laterality			
Right side	110 (49.5%)	3 (16.7%)	107 (52.4%)
Left side	95 (42.8%)	15 (83.3%)	80 (39.2%)
Bilateral	17 (7.7%)	0 (0.0%)	17 (8.3%)
MGMT			
Hypermethylated	109 (49.1%)	15 (83.3%)	94 (46.1%)
Unmethylated	101 (45.5%)	1 (5.6%)	100 (49.0%)
Missing	12 (5.4%)	2 (11.1%)	10 (4.9%)
IDH			
Wt	213 (95.9%)	17 (94.4%)	196 (96.1%)
Missing	9 (4.1%)	1 (5.6%)	8 (3.9%)
Comorbidities	132 (59.5%)	10 (55.6%)	122 (59.8%)

Data are presented as mean ± standard deviation, median (range) and number of observations, or number (percentage).

MGMT, ^6^O-methylguanine-DNA methyltransferase; IDH, isocitrate dehydrogenase; wt, wildtype.

### 3.2. Treatment characteristics

The treatment characteristics are shown in Table 2. Of the 222 patients, 117 (52.7%) underwent CRET, 76 (34.2%) underwent incomplete resection, 29 (13.1%) had biopsy. 206 (92.8%) patients received postoperative oncological treatment. The median time from surgery to start of oncological treatment was 38 days. A total of 125 (56.3%) patients received both radiotherapy and chemotherapy as primary oncological treatment. Of these, 109 (49.1%) initiated radiotherapy with 60 Gy in 30 fractions with concomitant TMZ (75 mg/m^2^) followed by adjuvant TMZ (200 mg/m^2^ 5 days every 4 week). In 40 (18.0%) patients, radiotherapy alone was provided, while in three cases radiotherapy was not completed. In those completing radiotherapy as monotherapy, the majority, 32 of 37 patients received 34 Gy. There were 41 (18.5%) patients that received chemotherapy alone, with TMZ being the chemotherapy of choice in 40 patients.

**Table 2:** Treatment characteristics.

Variable	Total *N* = 222	Alive *N* = 18	Dead *N* = 204
Operation/biopsy			
CRET	117 (52.7%)	14 (77.8%)	103 (50.5%)
Incomplete resection	76 (34.2%)	3 (16.7%)	73 (35.8%)
Biopsy	29 (13.1%)	1 (5.6%)	28 (13.7%)
Postoperative complications	29 (13.1%)	0 (0.0%)	29 (14.2%)
Start of oncological treatment (days after operation)	39 ± 14	37 ± 10	39 ± 14
	38 (13–82)	37 (15–60)	38 (13–82)
	*n* = 206	*n* = 18	*n* = 188
Duration of oncological treatment (days)	150 ± 154	299 ± 376	136 ± 104
	145 (2–1,761)	229 (15–1,761)	138 (2–453)
	*n* = 206	*n* = 18	*n* = 188
Oncological treatment			
Radiotherapy + chemotherapy^a^	125 (56.3%)	16 (88.9%)	109 (53.4%)
Chemoradiotherapy and adjuvant TMZ	109 (49.1%)	15 (83.3%)	94 (46.1%)
Short-course radiotherapy + TMZ	12 (5.4%)	1 (5.6%)	11 (5.4%)
Radiotherapy alone	40 (18.0%)	2 (11.1%)	38 (18.6%)
Chemotherapy alone	41 (18.5%)	0 (0.0%)	41 (20.1%)
Oncological treatment group incl MGMT promoter methyaltion			
Other than chemoradiotherapy and adjuvant TMZ	113 (52.1%)	3 (18.8%)	110 (54.7%)
Chemoradiotherapy and adjuvant TMZ/+MGMT^b^/+CRET^c^	35 (16.1%)	10 (62.5%)	25 (12.4%)
Chemoradiotherapy and adjuvant TMZ/+MGMT	20 (9.2%)	2 (12.5%)	18 (9.0%)
Chemoradiotherapy and adjuvant TMZ/+CRET	38 (17.5%)	1 (6.3%)	37 (18.4%)
Chemoradiotherapy and adjuvant TMZ/‒MGMT^d^/‒CRET^e^	11 (5.1%)	0 (0.0%)	11 (5.5%)
Treatment at recurrence	117 (52.7%)	11 (61.1%)	106 (52.0%)
Days from operation to recurrence treatment	371 ± 224	483 ± 229	359 ± 221
	306 (98–1,395)	463 (197–856)	305 (98–1,395)
	*n* = 117	*n* = 11	*n* = 106
Reoperation	30 (13.5%)	4 (22.2%)	26 (12.7%)
Days from operation to reoperation	518 ± 354	753 ± 160	482 ± 363
	424 (1–1,401)	777 (546–913)	339 (1–1,401)
	*n* = 30	*n* = 4	*n* = 26
Days from operation to death/censoring	504 ± 396	1,284 ± 427	435 ± 310
	392 (20–1,899)	1,204 (731–1,899)	371 (20–1,666)
	*n* = 222	*n* = 18	*n* = 204

Data are presented as mean ± standard deviation, median (range) and number of observations, or number (percentage).

CRET, complete resection of enhancing tumor; TMZ, temozolomide; MGMT, ^6^O-methylguanine-DNA methyltransferase.

^a^ Radiotherapy + Chemotherapy: given either concomitantly or sequentially, including also RT with concomitant and adjuvant TMZ, and short-course radiotherapy + TMZ.

^b^ +MGMT, hypermethylated MGMT promoter.

^c^ +CRET, operated with CRET.

^d^ –MGMT, unmethylated MGMT promoter.

^e^ –CRET, incomplete resection or biopsy.

There were 30 (13.5%) patients undergoing reoperation at recurrence. Of these, 6 had a third surgery. The median time between first operation and reoperation was 424 days. Oncological treatment at recurrence was provided in 117 (52.7%) patients. The most frequent treatments at first recurrence were: Lomustine (49 patients; 22.1%) and TMZ (48 patients; 21.6%), other rare options were PCV, Bevacizumab, Irinotecan, Carboplatin-Etoposid and Nivolumab. A total of 7 (3.2%) patients received re-irradiation.

### 3.3. Survival

**Figure 2:**
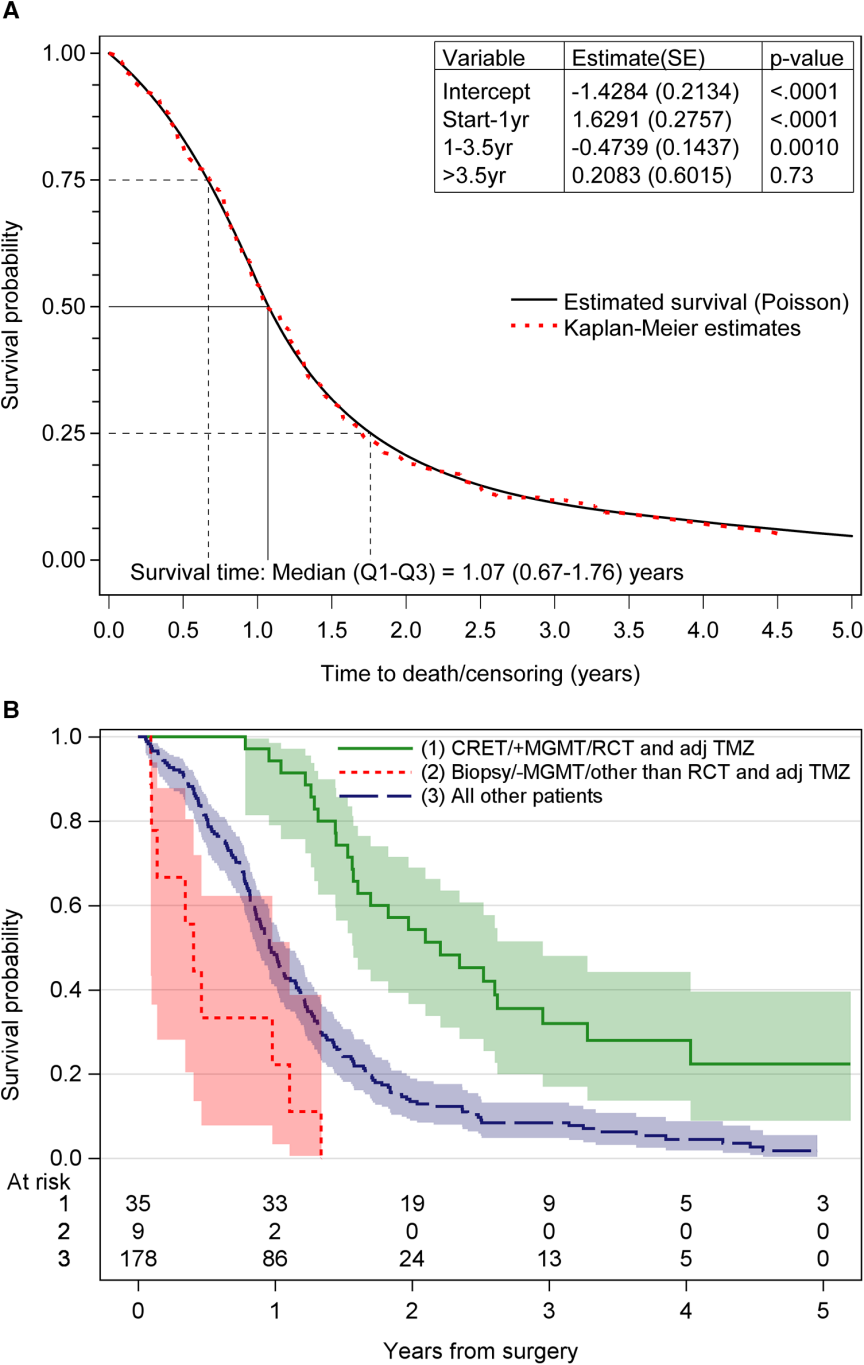
(**A**) The overall survival probability obtained by poisson regression for time-varying data and Kaplan–Meier technique. (**B**) Survival probability for the different groups of patients. CRET, complete resection of enhancing tumor; MGMT, ^6^O-methylguanine-DNA methyltransferase; RCT and adj TMZ, postoperative radiochemotherapy and adjuvant temozolomide; +MGMT, methylated MGMT promoter; –MGMT, unmethylated MGMT promoter.

The median OS for the study population was 1.07 years. Survival rates after surgery were 82% at 6 months, 55% at 12 months and 19% at 24 months. At the end of the study, 18 patients (8.1%) were alive. For patients treated with postoperative chemoradiotherapy and adjuvant TMZ, the median OS was 19.5 months. Figure 2A shows the survival for all patients. Figure 2B shows the survival probability for the patients with the most and least favorable combination of treatment- and tumor-related factors, compared to all other patients.

### 3.4. Univariable analysis

Table 3 shows the age- and sex-adjusted survival estimates of the variables, including significant interactions with time. As shown, age was strongly associated to survival, each additional year in age represented a 3% increase in the risk of death. Patients with central tumor component, temporal tumor component, bilateral tumor location, right-sided tumor location, multilobular tumor also had an increased risk of death. In contrast, good performance status, no comorbidities, CRET and MGMT promoter methylation were associated with better prognosis. There were significant interactions with time for the variables central tumor location (HR: 0.52, meaning that the increased risk of the central tumors decreases with time) and oncological treatment (HR: 0.01, meaning that the decreased risk of the oncological treatment decreases further with time). In the sub-analysis of the different treatment modalities, we found significantly longer survival for the 109 patients receiving postoperative chemoradiotherapy and adjuvant TMZ compared to the other modalities. Of these, patients with hypermethylated MGMT promoter had significantly better prognosis, with a 63% lower risk of death.

**Table 3:** Age- and sex-adjusted survival analysis using extended poisson regression including significant interactions with time.

Predictor	Value	*n* (%) events	event rate (95% CI) per 10 person-years	Hazard ratio (95% CI)	*p*-value
Sex	Male	126 (90.6%)	6.55 (5.45–7.80)		
	Female	78 (94.0%)	6.83 (5.40–8.52)	1.16 (0.87–1.54)	0.32
Age at operation	Per 1 year increase	204 (91.9%)	6.65 (5.77–7.63)	1.03 (1.01–1.04)	0.0001
Age at operation	<50	26 (89.7%)	4.73 (3.09–6.93)		
	50-<60	51 (87.9%)	5.16 (3.84–6.78)	1.07 (0.67–1.72)	0.77
	60-<70	82 (93.2%)	8.03 (6.39–9.97)	1.83 (1.17–2.87)	0.0083
	≥70	45 (95.7%)	8.87 (6.47–11.86)	2.08 (1.28–3.41)	0.0034
Epileptic seizures	Yes	52 (89.7%)	5.14 (3.84–6.75)	0.73 (0.53–1.00)	0.05
	No	152 (92.7%)	7.39 (6.27–8.67)		
Headache	Yes	85 (94.4%)	6.86 (5.48–8.48)	1.27 (0.94–1.73)	0.12
	No	119 (90.2%)	6.51 (5.39–7.79)		
Nausea/vomiting	Yes	37 (97.4%)	7.07 (4.98–9.74)	1.27 (0.87–1.85)	0.21
	No	167 (90.8%)	6.57 (5.61–7.64)		
Personality change	Yes	38 (92.7%)	7.67 (5.43–10.53)	1.29 (0.90–1.84)	0.16
	No	166 (91.7%)	6.46 (5.51–7.52)		
Psychological reactions and symptoms	Yes	13 (92.9%)	7.93 (4.22–13.56)	1.56 (0.88–2.77)	0.13
	No	191 (91.8%)	6.58 (5.68–7.58)		
Focal symptoms	Yes	129 (90.8%)	6.60 (5.51–7.85)	0.92 (0.69–1.22)	0.56
	No	75 (93.8%)	6.74 (5.30–8.45)		
WHO performance status	WHO 2/3/4	62 (93.9%)	9.26 (7.10–11.87)	1.60 (1.16–2.20)	0.0043
	WHO 0/1	142 (91.0%)	5.92 (4.99–6.98)		
Frontal	Yes	81 (93.1%)	6.96 (5.53–8.65)	1.03 (0.77–1.36)	0.85
	No	123 (91.1%)	6.46 (5.37–7.71)		
Temporal	Yes	108 (93.9%)	7.82 (6.41–9.44)	1.45 (1.10–1.92)	0.0088
	No	96 (89.7%)	5.70 (4.61–6.96)		
Parietal	Yes	68 (91.9%)	6.75 (5.24–8.56)	1.06 (0.79–1.42)	0.70
	No	136 (91.9%)	6.60 (5.54–7.81)		
Occipital	Yes	33 (91.7%)	6.52 (4.49–9.15)	0.94 (0.65–1.37)	0.74
	No	171 (91.9%)	6.68 (5.72–7.76)		
Central	Yes	55 (93.2%)	9.14 (6.89–11.90)	3.46 (1.95–6.13)	<.0001
Interaction: (Time)*(Central)				0.52 (0.32–0.85)	0.0087
	No	149 (91.4%)	6.05 (5.11–7.10)		
Side	Right side	107 (97.3%)	7.66 (6.28–9.25)		
	Left side	80 (84.2%)	5.14 (4.08–6.40)	0.64 (0.47–0.85)	0.0025
Bilateral	Yes	17 (100.0%)	14.93 (8.69–23.90)	3.01 (1.81–5.02)	<.0001
	No	187 (91.2%)	6.33 (5.46–7.31)		
Multifocal	Yes	49 (92.5%)	7.88 (5.83–10.41)	1.34 (0.97–1.84)	0.08
	No	155 (91.7%)	6.34 (5.38–7.42)		
Multilobular	Yes	100 (92.6%)	7.64 (6.22–9.30)	1.33 (1.01–1.76)	0.041
	No	104 (91.2%)	5.92 (4.83–7.17)		
Number of lobes involved	1	104 (91.2%)	5.92 (4.83–7.17)	1.50 (1.25–1.81)	<.0001
	2	65 (89.0%)	6.17 (4.76–7.87)		
	3	30 (100.0%)	12.97 (8.75–18.51)		
	4	4 (100.0%)	21.24 (5.79–54.37)		
	5	1 (100.0%)	18.45 (0.47–102.78)		
Surgical treatment	CRET	103 (88.0%)	5.40 (4.41–6.55)		
	Incomplete resection vs. CRET	73 (96.1%)	8.47 (6.64–10.65)	1.72 (1.27–2.33)	0.0004
	Biopsy vs. CRET	28 (96.6%)	9.42 (6.26–13.61)	1.82 (1.18–2.80)	0.0064
	Biopsy vs. Other	28 (96.6%)	9.42 (6.26–13.61)	1.06 (0.68–1.65)	0.81
Comorbidities	Yes	122 (92.4%)	8.06 (6.70–9.63)	1.37 (1.01–1.86)	0.045
	No	82 (91.1%)	5.28 (4.20–6.55)		
Number of medications	None	97 (91.5%)	5.58 (4.53–6.81)		
	1	28 (87.5%)	7.15 (4.75–10.33)		
	2	31 (96.9%)	8.64 (5.87–12.26)		
	3	20 (87.0%)	8.83 (5.39–13.64)		
	4	16 (94.1%)	6.71 (3.84–10.90)		
	≥5	12 (100.0%)	10.63 (5.49–18.57)	1.05 (0.97–1.15)	0.23
Hypermethylated MGMT promoter	Yes	94 (86.2%)	5.23 (4.22–6.40)	0.48 (0.35–0.64)	<.0001
	No	100 (99.0%)	9.24 (7.52–11.24)		
Oncological treatment [assumed from baseline (immortal time bias)]	Yes	188 (91.3%)	6.19 (5.34–7.14)	0.04 (0.02–0.07)	<.0001
	No	16 (100.0%)	55.08 (31.48–89.45)		
Oncological treatment (time-updated)				0.49 (0.20–1.19)	0.11
Interaction: (Time)*[Oncologic treatment (time-updated)]				0.01 (0.00–0.13)	0.0010
No oncological treatment vs. chemoradiotherapy and adjuvant TMZ				11.49 (5.24–25.21)	<.0001
Short-course RT + TMZ vs. chemoradiotherapy and adjuvant TMZ				2.20 (1.15–4.21)	0.018
RT alone vs. chemoradiotherapy and adjuvant TMZ				3.22 (2.03–5.11)	<.0001
TMZ alone vs. chemoradiotherapy and adjuvant TMZ				4.41 (2.76–7.04)	<.0001
Chemoradiotherapy and adjuvant TMZ w vs. wo MGMT hypermethylation	Chemoradiotherapy and adjuvant TMZ w MGMT	43 (78.2%)	3.38 (2.44–4.55)	0.37 (0.24–0.57)	<.0001
	Chemoradiotherapy and adjuvant TMZ wo MGMT	48 (98.0%)	6.71 (4.95–8.90)		

CRET, complete resection of enhancing tumor; MGMT, ^6^O-methylguanine-DNA methyltransferase; RT, radiotherapy; TMZ, temozolomide.

### 3.5. Multivariable analysis

The following variables, listed in order of importance (Table 4), were identified as independent predictors for longer survival: MGMT promoter hypermethylation (vs. unmethylated MGMT promoter), non-central tumor location including the interaction term with time, CRET (vs. no CRET), WHO PS 0–1 (vs. WHO PS 2–4), unilateral tumor location, one lobe involved, younger age and no comorbidities.

**Table 4:** Significant multivariable model including independent predictors using extended poisson regression models for time to death.

Predictor	Value	Hazard ratio (95% CI)	Standardized hazard Ratio (95% CI) per 1 SD	*p*-value	Importance
Age at operation	Per 1 unit increase	1.02 (1.01–1.04)	1.26 (1.06–1.50)	0.0085	7
MGMT methylation	Yes vs. No	0.37 (0.27–0.50)	0.61 (0.52–0.71)	<.0001	1
WHO performance status	WHO 2/3/4 vs. 0/1	1.82 (1.31–2.53)	1.32 (1.13–1.53)	0.0004	4
Location	Central vs. Other	2.23 (1.09–4.56)	1.42 (1.04–1.95)	0.027	2
	Interaction: (Time)*(Central vs. Other)	0.58 (0.34–0.99)	0.79 (0.62–1.00)	0.046	2
Bilateral	Yes vs. No	2.50 (1.28–4.91)	1.28 (1.07–1.54)	0.0077	5
Number of lobes involved	Per 1 unit increase	1.34 (1.06–1.70)	1.27 (1.05–1.53)	0.013	6
Surgical treatment	Incomplete resection vs. CRET	1.88 (1.36–2.59)	1.35 (1.16–1.58)	0.0001	3
	Biopsy vs. CRET	1.38 (0.77–2.48)	1.12 (0.92–1.36)	0.28	
Comorbidities	Yes vs. No	1.51 (1.07–2.12)	1.22 (1.03–1.45)	0.019	8

Time since operation modelled as continuous with break points at 1 and 3.5 years.

For MGMT No vs. Yes gives: unstandardized HR: 2.70 (2.00–3.70), standardized HR: 1.64 (1.41–1.92).

MGMT, ^6^O-methylguanine-DNA methyltransferase; CRET, complete resection of enhancing tumor; HR, hazard ratio.

### 3.6. Comparison of the two cohorts

**Figure 3:**
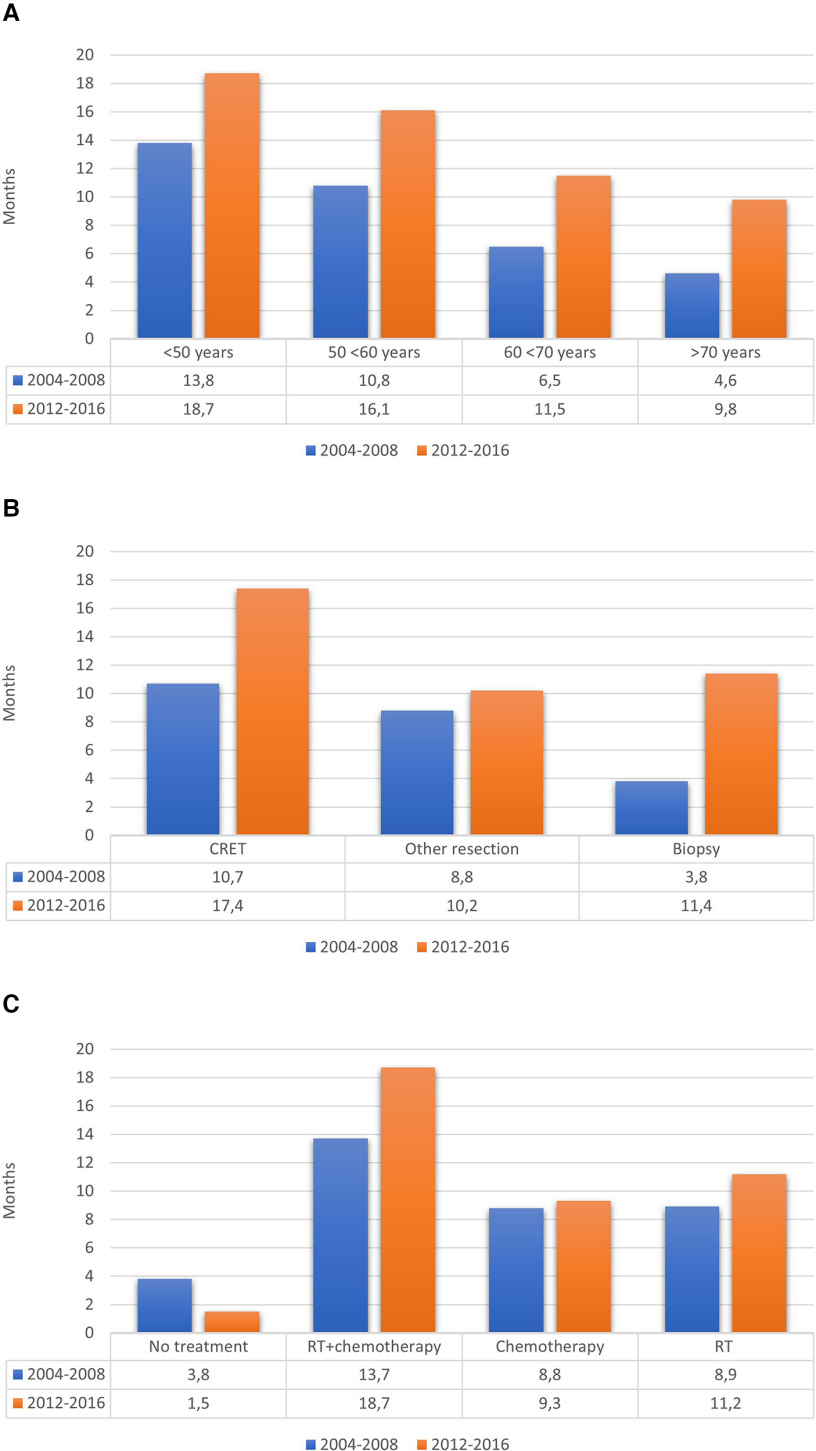
Median OS in relation to (**A**) age, (**B**) surgical treatment and (**C**) post-operative oncological treatment.

Table 5 shows the comparison of main characteristics between the two cohorts. The OS in the cohort from 2004 to 2008 was 0.73 years (8.8 months), compared to 1.07 years (12.8 months) in the cohort from 2012 to 2016, including the 25 patients with IDH-mutated tumors. Survival rates at 6, 12 and 24 months were respectively 65%, 36%, 11% in the previous cohort vs. 84%, 57%, 24% in the new cohort. Figure 3 shows the differences regarding median OS in relation to age, surgical treatment and post-operative oncological treatment between the two cohorts.

**Table 5:** Comparison of patient characteristics, tumor characteristics and treatment characteristics between our two cohorts.

Variable	2004–2008 *N* = 229	2012–2016 *N* = 247	*p*-value
Male	136 (59.4%)	150 (60.7%)	0.78
Age at operation (years)	58.6 ± 10.7	60.1 ± 12.3	0.039
	60 (22–82)	62 (19–82)	
Age at operation (years) (cat.)			0.11
<50	47 (20.5%)	45 (18.2%)	
50-<60	68 (29.7%)	63 (25.5%)	
60-<70	83 (36.2%)	92 (37.2%)	
≥70	31 (13.5%)	47 (19.0%)	
Multifocal	56 (24.5%)	57 (23.1%)	0.75
Bilateral	27 (11.8%)	18 (7.3%)	0.12
Right side	97 (48.0%)	119 (52.0%)	0.44
Operation/biopsy^a^			0.36
Radical/CRET	122 (53.3%)	131 (53.0%)	
Partial/Incomplete resection	71 (31.0%)	87 (35.2%)	
Biopsy	36 (15.7%)	29 (11.7%)	
Oncological treatment			<.0001
None	79 (34.5%)	16 (6.5%)	
Radiotherapy + Chemotherapy	95 (41.5%)	138 (55.9%)	
Chemotherapy only	17 (7.4%)	49 (19.8%)	
Radiotherapy only	38 (16.6%)	44 (17.8%)	
Reoperation	19 (8.3%)	36 (14.6%)	0.044
Dead	216 (94.3%)	221 (89.5%)	0.07
Months from surgery to death	11.7 ± 10.8	17.7 ± 14.0	<.0001
	8.9 (0.0–67.4)	13.9 (0.7–62.4)	

Data are presented as mean ± standard deviation, median (range) or number (percentage).

For difference between cohorts Fisher's exact test was used for dichotomous variables, *χ*^2^ test for non-ordered categorical variables and Mann–Whitney *U*-test for continuous variables.

CRET, complete resection of enhancing tumor.

^a^ Radicality determined by the surgeon in the retrospective study, while in the current study evaluated with postoperative MRI.

## 4. Discussion

Healthcare in Sweden for patients with complicated or rare diseases such as GBM is centralized at university hospitals. This allows population-based studies, ensuring data from all patients presenting with the disease within a defined time period. Here, we present data on the clinical management and outcome in patients operated for GBM during 2012–2016. We found a significant change towards more active surgery and multimodal treatment over time with consequent increased survival, in comparison with a corresponding study from 2004 to 2008. Of the established prognostic markers, MGMT promoter hypermethylation was the strongest predictor for longer survival in our cohort. Other independent variables for prognosis were tumor location, extent of surgery, PS, age and comorbidities.

These findings are all consistent with earlier studies, including large, randomized trials that have provided accumulating evidence for the predictive value of MGMT promoter methylation in GBM (14, 28–31). It is important to note that there may have been synergism between various favourable treatment-related factors in our material. This is reflected by the long median OS of 2.21 years in the subgroup who had CRET, postoperative chemoradiotherapy and adjuvant TMZ, and hypermethylated MGMT promoter, in which more than 10% of patients survived 4 years or longer. It is obvious from our data and previously published studies that small subgroups of patients exist with GBM who will be long-term survivors. In contrast, central tumor location and bilateral tumor location were identified as independent predictors for poor survival. Similar findings have been shown in earlier studies (4, 7, 10, 32, 33). Fyllingen et al. for example reported reduced OS in patients with centrally located tumors, and suggested the actual distance from the center of third ventricle to the contrast-enhancing tumor as a possible prognostic factor for survival (34). A nuance in our study was that for patients with central tumor location, there was a significant interaction with time. This statistical interaction means that the value of HR changes over time. In other words, patients with a central tumor component have a significantly higher risk of death at the time of diagnosis than those with non-central tumor location, but this risk gradually decreases over time.

Regarding the role of surgery, MRI-defined CRET was a strong independent prognostic factor in our cohort, which is in line with previous studies, especially in post-TMZ era (6, 9, 22, 35). Of interest in this context, and corroborating earlier studies in GBM IDH-wt with data on MGMT methylation status, is the fact that the prognostic benefit of the incomplete resection was not different from biopsy (36, 37).The role of the extent of surgery and its effect on survival are controversial, with those proposing precise thresholds for the extent of resection to achieve a significant effect on survival, while others suggest a continuous relationship between the extent of surgery and survival (38).

Another expected finding was the favourable prognostic effect of good PS, confirming previous population-based studies (4, 21, 22, 29). We recorded PS at the time point before surgery, and found a similar distribution of the different WHO PS grades for these patients as reported by Hansen et al. (4).

Age was another strong prognostic factor, corroborating that younger age in patients with GBM is an independent prognostic factor for survival (4, 20, 39, 40).

Approximately 50% of the patients initiated the full multimodal treatment regime in our cohort with postoperative chemoradiotherapy and adjuvant TMZ, comparable with the population-based studies by Hansen et al. (50%) and Eriksson et al. (55%) (4, 22). The median OS for these patients was 19.5 months, i.e., somewhat higher than 16.2 months and 16.4 months reported in similar population-based settings (19, 29).

### 4.1. A gradual improvement in care

The secondary goal of this study was to investigate changes in outcome and clinical management over time. For this purpose, we compared the obtained data with those from a previously published cohort at our hospital. Although the inclusion criteria in both studies were similar, there were some differences in study designs, requiring carefulness in the interpretation of the results. As highlighted by Preusser et al., using historical controls strongly limits the comparability of data between studies with differences in inclusion criteria or study design (41). Another major difference is that in the current study, the extent of resection was evaluated by MRI, but evaluated by the operating surgeon in the historical cohort. Comparing data from several studies, Woehrer et al. describes a survival gain of about 1–2 months after the introduction of TMZ in the first line treatment (42). Postoperative oncological treatment with chemoradiotherapy and adjuvant TMZ was introduced in 2006 in our region, and successively implemented during the following years. Our hypothesis was that implementing this treatment contributed to longer survival in the population of GBM patients in our region. Compared to our previous study (2004–2008), there was indeed a significant increase in OS for patients with GBM treated according to current standard treatment (0.73 years = 8.8 months vs. 1.07 years = 12.8 months). These data are comparable to the result of a Swedish study by Bruhn et al. who reported a median OS of 8.6 months before vs. 12.3 months after the introduction of postoperative chemoradiotherapy and adjuvant TMZ (20). Other population-based studies from the post-TMZ era reported a slightly lower median OS; between 10.0 and 11.8 months (4, 19, 21, 22, 29, 40, 43).

It is, however, unlikely that the change in postsurgical treatment strategies is the only explanation for the increased OS for patients with GBM. Indeed, the proportion of patients with suspected GBM not selected for surgery has steadily decreased (46.7% vs. 34.8%) since our previous study, reflecting a more optimistic surgical attitude, while at the same time, the number of patients undergoing surgery has increased for each year. Thus, there was a clear trend in our region towards older patients being accepted for surgery compared to previous years. The proportion of operated patients over 70 years old was 19.0%, compared to 13.5% in the previous study. Interestingly, a numerically longer median OS was present for all age categories (Figure 3A), suggesting that the positive effect of a more active surgical approach was independent of biological age.

The proportion of patients that underwent complete resection was about the same in the previous retrospective study as in the recent study (53.3% and 53.0%, respectively). The high percentage of radically operated patients in the retrospective study may be explained by the fact that the extent of surgery was estimated by the surgeon, while in the recent study the assessment of radicality was based on postoperative MRI. By this approach, a larger proportion of patients underwent partial surgery/incomplete resection (35.2% vs. 31.0%) and a smaller proportion of patients underwent biopsy (15.7% vs. 11.7%).

To conclude, and as clearly illustrated in Figure 3B, the median OS for patients with GBM in recent years has increased in all groups, regardless of the extent of resection. There may be several factors explaining the increased OS: the surgical as well the imaging techniques have improved, and more patients have received postoperative oncological treatment. In the current study, considerably more patients initiated both radio- and chemotherapy, 55.9% vs. 41.5%. Furthermore, the proportion of patients who did not receive any postoperative oncological treatment has decreased significantly (34.5% vs. 6.5%) over time. Compared to the studies by Fabbro-Peray et al., Hansen et al. and Graus et al., this proportion was low (6.5% vs. 20%, 22% respectively 22.3%) (4, 21, 43). There was also a tendency to give radiotherapy or chemotherapy as single treatment to patients among those who have not been considered suitable for chemoradiotherapy.

### 4.2. Strengths and limitations

The main strength of our study is that it is population-based, with high external validity. We were able to collect almost complete data for MGMT promoter methylation status. Although data were prospectively collected, the analysis was of retrospective character, which should be regarded as a limitation. Consequently, we were not always able to register if patients actually completed the prescribed oncological treatment.

## 5. Conclusion

MGMT promoter methylation status, followed by non-central tumor location and CRET, were the strongest prognostic factors for survival in this population-based study of patients with GBM. We found a significant change towards more active treatment strategies over time with consequent increased survival for patients with GBMs in our region, especially in the best prognostic group.

## Data Availability

The original contributions presented in the study are included in the article/Supplementary Material, further inquiries can be directed to the corresponding author.
